# Correction: Luongo et al. Cannabidiol and Oxygen-Ozone Combination Induce Cytotoxicity in Human Pancreatic Ductal Adenocarcinoma Cell Lines. *Cancers* 2020, *12*, 2774

**DOI:** 10.3390/cancers17030365

**Published:** 2025-01-23

**Authors:** Margherita Luongo, Oliviero Marinelli, Laura Zeppa, Cristina Aguzzi, Maria Beatrice Morelli, Consuelo Amantini, Andrea Frassineti, Marianne di Costanzo, Alessandro Fanelli, Giorgio Santoni, Massimo Nabissi

**Affiliations:** 1 “Maria Guarino” Foundation—AMOR No Profit Association, 80078 Pozzuoli, Italy; margherita.luongo@aslnapoli2nord.it (M.L.); info@dottorfrassineti.it (A.F.); marianne.dicostanzo@ospedalideicolli.it (M.d.C.); 2 School of Pharmacy, University of Camerino, 62032 Camerino, MC, Italy; oliviero.marinelli@unicam.it (O.M.); laura.zeppa@studenti.unicam.it (L.Z.); cristina.aguzzi@studenti.unicam.it (C.A.); mariabeatrice.morelli@unicam.it (M.B.M.); giorgio.santoni@unicam.it (G.S.); 3 School of Bioscience and Veterinary Medicine, University of Camerino, 62032 Camerino, MC, Italy; consuelo.amantini@unicam.it; 4 Department of Radiotherapy, Ecomedica Empoli, 50053 Empoli, FI, Italy; alessandro.fanelli@ecomedica.it; 5 Integrative Therapy Discovery Lab, University of Camerino, 62032 Camerino, MC, Italy

## Text Correction—Conflicts of Interest Statement

There was an error in the original publication [1]. A correction has been made to the Conflicts of Interest statement: M.N. declares that he is the CEO of Integrative Therapy Discovery Lab.

The authors state that the scientific conclusions are unaffected. This correction was approved by the Academic Editor. The original publication has also been updated.
