# Supplementary material for: Cannabidiol and Oxygen-Ozone Combination Induce Cytotoxicity in Human Pancreatic Ductal Adenocarcinoma Cell Lines
Source: Cancers (Basel). 2020 Sep 27;12(10):2774. doi: 10.3390/cancers12102774 (PMC7600087; doi:10.3390/cancers12102774)
Supplement: Supplementary file 1 [file cancers-12-02774-s001.pdf]

Supplementary Material: Cannabidiol and Oxygen-Ozone Combination Induce Cytotoxicity in Human Pancreatic Ductal Adenocarcinoma Cell Lines

Margherita Luongo, Oliviero Marinelli, Laura Zeppa, Cristina Aguzzi, Maria Beatrice Morelli, Consuelo Amantini, Andrea Frassineti, Marianne di Costanzo, Alessandro Fanelli, Giorgio Santoni and Massimo Nabissi

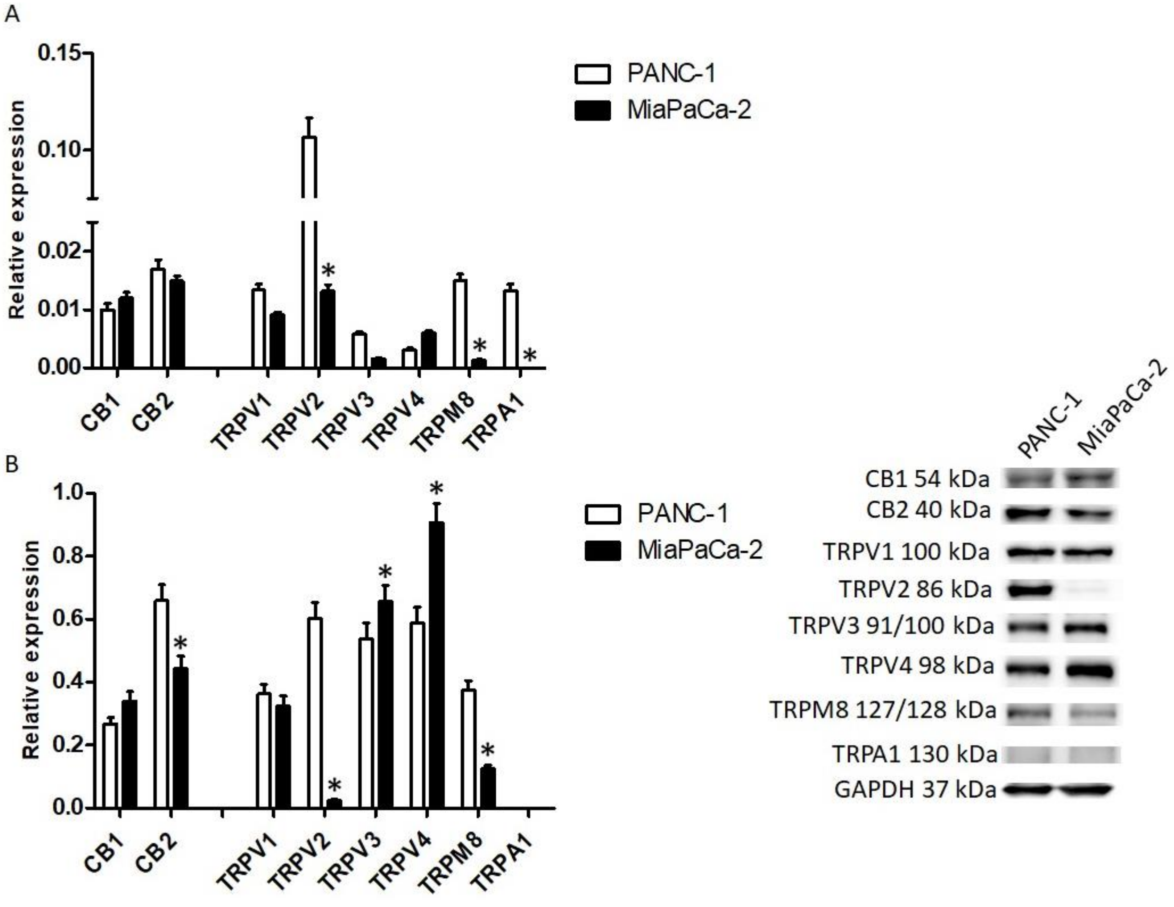

Figure S1. CBs and TRPs expression in PDAC cell lines. (**A**) CB1, CB2, TRPV1, TRPV2, TRPV3, TRPV4, TRPM8 and TRPA1 mRNA expression was evaluated by qRT-PCR in PANC-1 and MiaPaCa-2 cell lines. Target mRNA levels were normalized for GAPDH expression. Data are expressed as fold mean ± SE. * $p < 0.05$ PANC-1 vs MiaPaCa-2 cells. (**B**) CB1, CB2, TRPV1, TRPV2, TRPV3, TRPV4, TRPM8 and TRPA1 protein expression was evaluated by western blot in PANC-1 and MiaPaCa-2 cell lines. Full western blot images can be found in Figure S11. Densitometric values were normalized to GAPDH used as loading control. Densitometric values shown are the mean ± SE of three separate experiments. * $p < 0.05$ PANC-1 vs. MiaPaCa-2 cells.

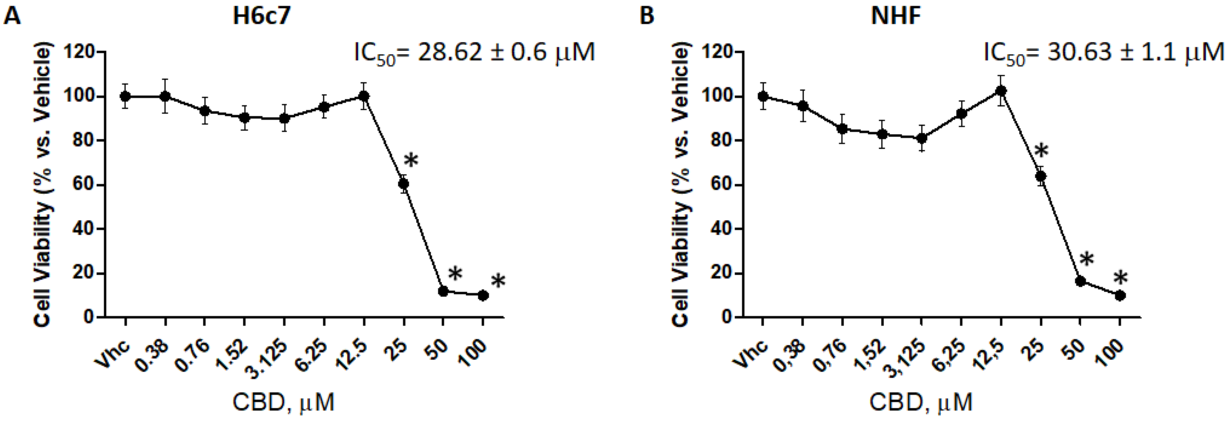

Figure S2. Human Pancreatic Duct Epithelial and Human Fibroblasts cell lines were more resistant to CBD. Cell viability was determined by MTT assay. (**A**) H6c7 and (**B**) NHF cells were treated for 72 h with different concentrations of CBD (up to 100 µM), in daily administration. Data shown are expressed as mean ± SE of three separate experiments. * $p < 0.05$ treated vs. vehicle.

Normalized Isobologram for Combo: cbdgem (CBD+GEM)

PANC-1

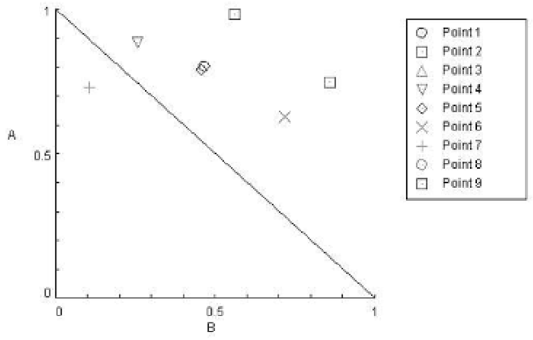

CI Data for Non-Constant Combo: cbdgem (CBD+GEM)

Dose CBD	Dose GEM	Effect	CI
25.0	100.0	0.16	1.26813
12.5	100.0	0.5	1.60814
6.25	100.0	0.69	1.66235
25.0	50.0	0.2	1.14556
12.5	50.0	0.54	1.24987
6.25	50.0	0.8	1.34724
25.0	25.0	0.13	0.83838
12.5	25.0	0.86	1.89625
6.25	25.0	0.93	1.54556

Normalized Isobologram for Combo: CBDPTX (CBD+PTX)

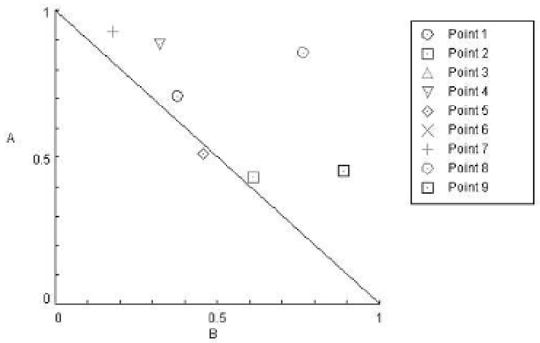

CI Data for Non-Constant Combo: CBDPTX (CBD+PTX)

Dose CBD	Dose PTX	Effect	CI
25.0	7.0	0.12	1.08444
12.5	7.0	0.19	1.04465
6.25	7.0	0.29	1.26796
25.0	3.5	0.2	1.21105
12.5	3.5	0.27	0.97231
6.25	3.5	0.51	1.52913
25.0	1.75	0.22	1.10869
12.5	1.75	0.59	1.62279
6.25	1.75	0.63	1.34671

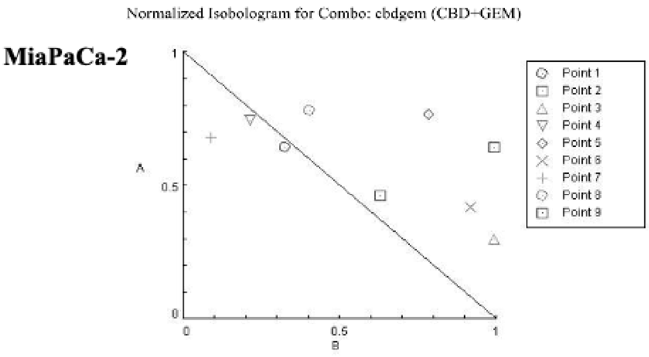

CI Data for Non-Constant Combo: cbdgem (CBD+GEM)

Dose CBD	Dose GEM	Effect	CI
25.0	100.0	0.08	0.96756
12.5	100.0	0.17	1.09374
6.25	100.0	0.27	1.29362
25.0	50.0	0.11	0.95975
12.5	50.0	0.4	1.55142
6.25	50.0	0.45	1.33759
25.0	25.0	0.09	0.76833
12.5	25.0	0.41	1.18559
6.25	25.0	0.69	1.63736

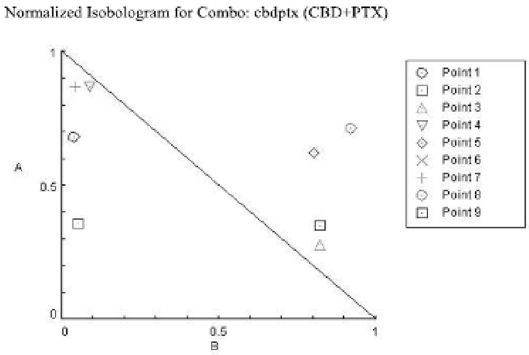

CI Data for Non-Constant Combo: cbdptx (CBD+PTX)

Dose CBD	Dose PTX	Effect	CI
25.0	7.0	0.09	0.71888
12.5	7.0	0.1	0.41122
6.25	7.0	0.24	1.10328
25.0	3.5	0.15	0.95990
12.5	3.5	0.29	1.42646
6.25	3.5	0.36	2.20411
25.0	1.75	0.15	0.91443
12.5	1.75	0.36	1.63685
6.25	1.75	0.35	1.17464

Figure S3. Isobologram plots and CI values for combination treatments of CBD, GEM and PTX in PANC-1 and MiaPaCa-2 cell lines. Isobologram plots for combination treatments of CBD, GEM and PTX in PANC-1 and MiaPaCa-2 cell lines. On the lower left of the hypotenuse synergism, on the hypotenuse additive effect, and on the upper right of the hypotenuse antagonism. Synergistic activity was assessed by CompuSyn software. CI values for combination treatments of CBD, GEM and PTX in PANC-1 and MiaPaCa-2 cell lines. CI = 1, < 1 and > 1 indicates additive effect, synergism and antagonism, respectively**.**

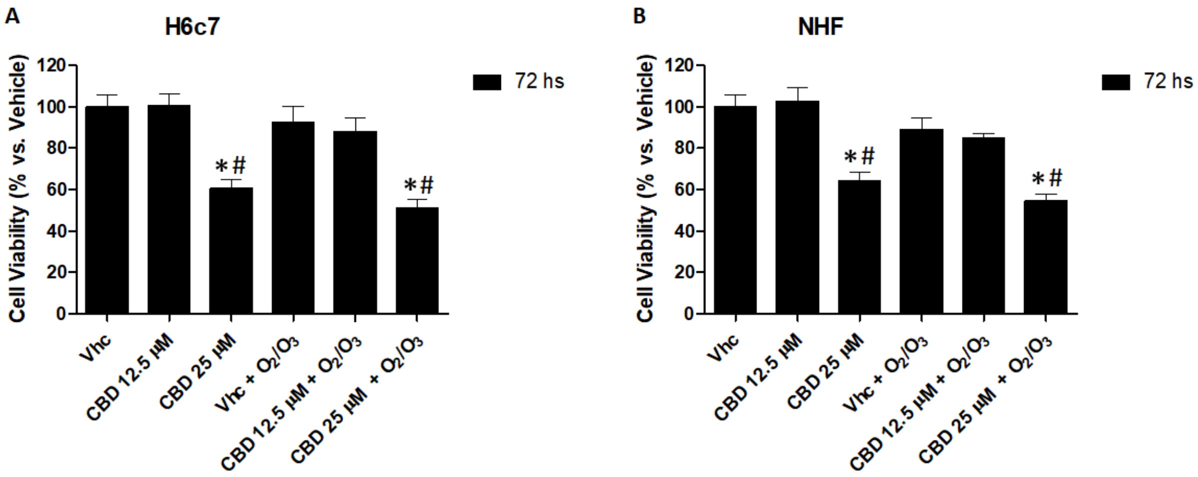

Figure S4. O_2/O_3 did not increase CBD cytotoxicity in H6c7 and NHF cell lines. Cell viability was determined by MTT assay. (**A**) H6c7 and (**B**) NHF cells were treated with O_2/O_3 and CBD (12.5 and 25 μM) in daily administration. Data shown are expressed as mean ± SE of three separate experiments. * $p < 0.05$ treated vs. vehicle, # $p < 0.05$ vs. CBD.

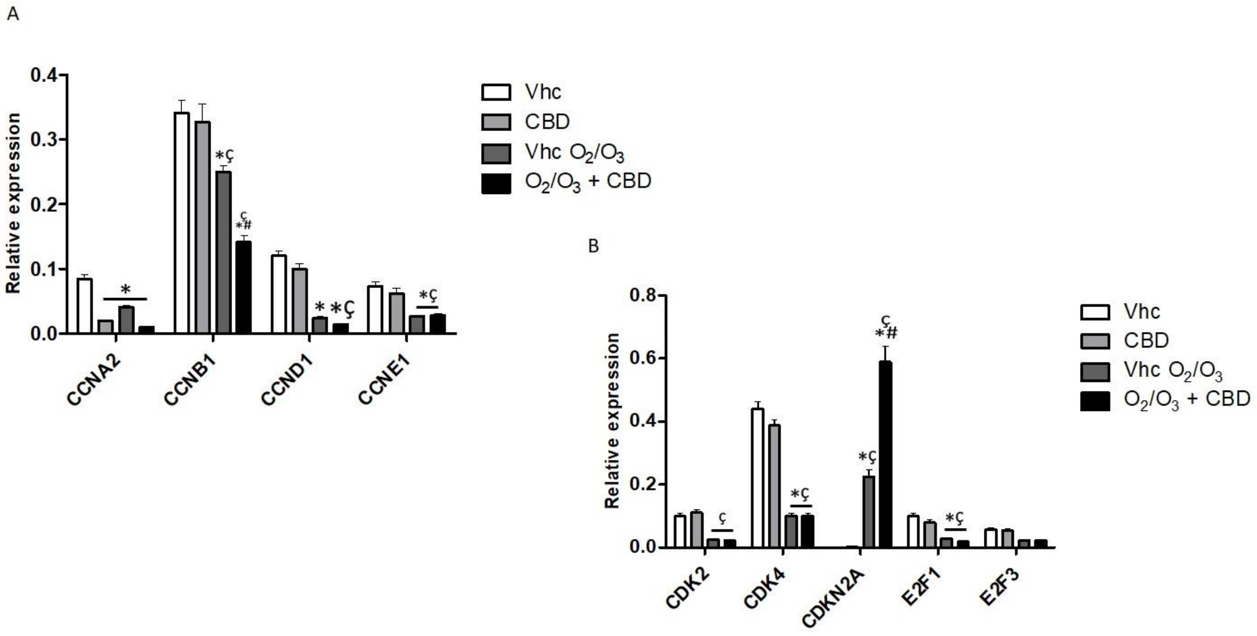

Figure S5. Modulation of cell cycle pathways in MiaPaCa-2 cell line. (**A**) CCNA2, CCNB1 and CCND1, CCNE1 mRNA expression and (**B**) CDK2, CDK4, CDKN2A, E2F1 and E2F3 mRNA expression was evaluated by qRT-PCR in MiaPaCa-2 cell line, treated with CBD in presence and absence of O_2/O_3. Target mRNA levels were normalized for GAPDH expression. Data are expressed as fold mean ± SE. * $p < 0.05$ vs. Vhc, #,* $p < 0.05$ vs. Vhc O_2/O_3, ç $p < 0.05$ vs. CBD.

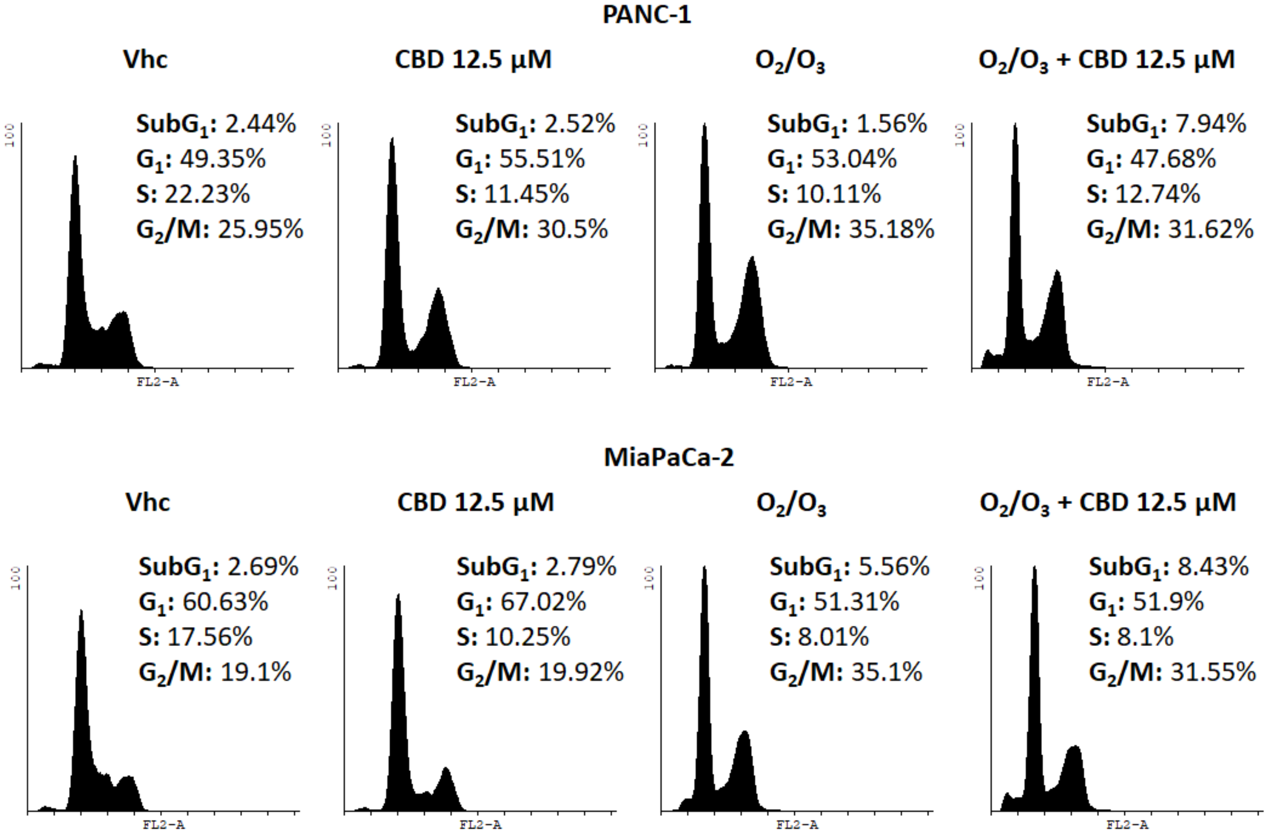

Figure S6. O2/O3, alone and in combination with CBD, reduced cell cycle progression in PANC-1 and MiaPaCa-2 cell lines. O_2/O_3, alone and in combination with CBD, reduced cell cycle progression in PANC-1 and MiaPaCa-2 cell lines. Cells treated with CBD (12.5 µM), in presence and absence of O2/O3, for 12 h. Representative cell cycle distribution by FACS analysis.

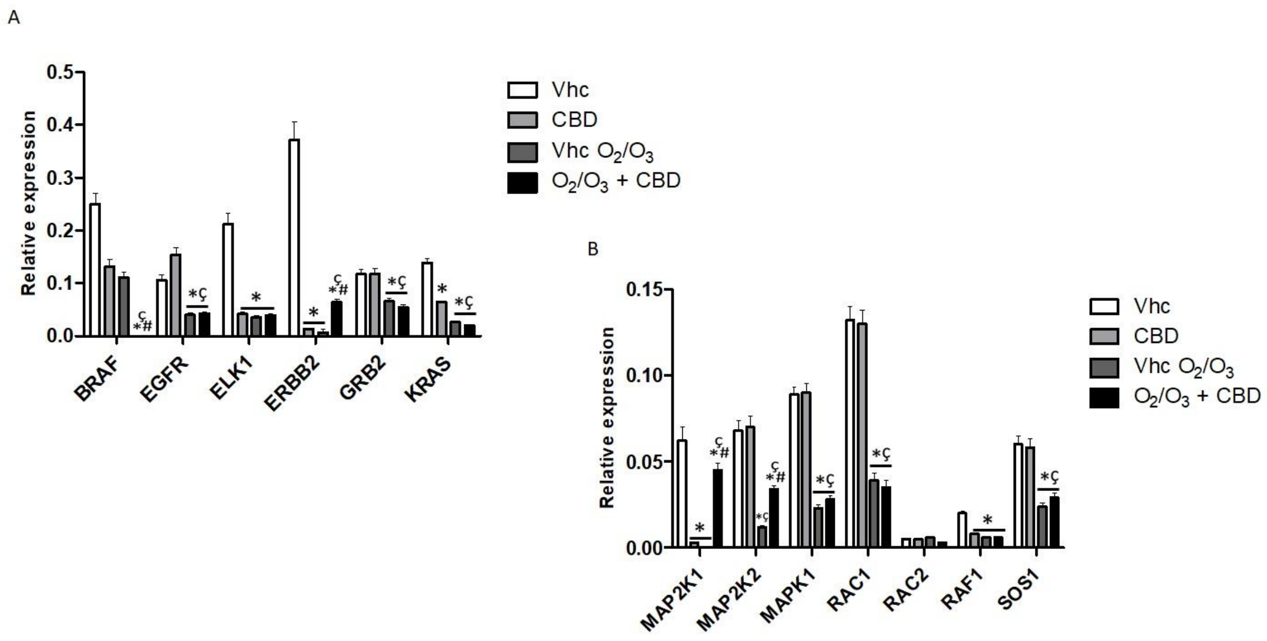

Figure S7. Modulation of Ras pathways in MiaPaca-2 cell line. (**A**) BRAF, EGFR, ELK1, ERBB2, GRB2 and KRAS mRNA expression and (**B**) MAP2K1, MAP2K2, MAPK1, RAC1, RAC2, RAF1 and SOS1 mRNA expression was evaluated by qRT-PCR in MiaPaCa-2 cell line, treated with CBD in presence and absence of O_2/O_3. Target mRNA levels were normalized for GAPDH expression. Data are expressed as fold mean ± SE. * $p < 0.05$ vs. Vhc, $^{\#,*}$ $p < 0.05$ vs. Vhc O_2/O_3, ^{ç} $p < 0.05$ vs. CBD.

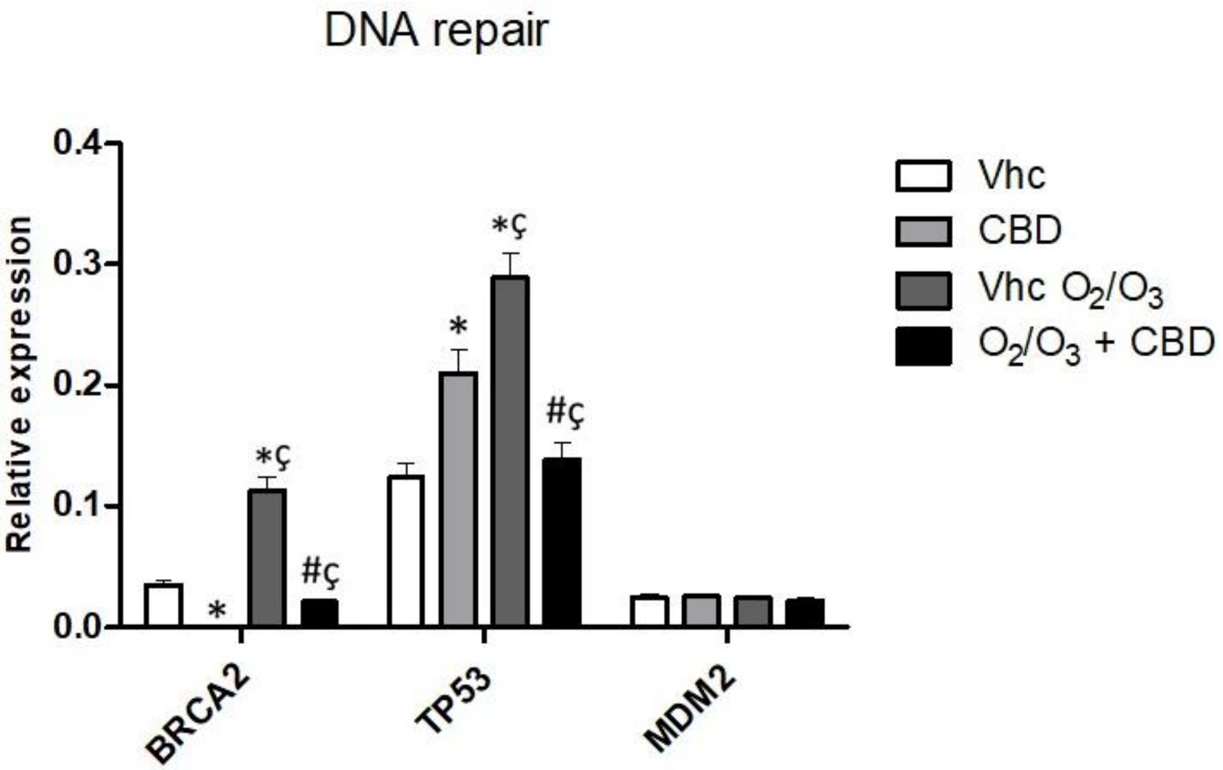

Figure S8. Modulation of DNA repair pathways in MiaPaCa-2 cell line. BRCA2, TP53 and MDM2 mRNA expression was evaluated by qRT-PCR in MiaPaCa-2 cell line, treated with CBD in presence and absence of O_2/O_3. Target mRNA levels were normalized for GAPDH expression. Data are expressed as fold mean ± SE. * $p < 0.05$ vs. Vhc, $^{\#,*}$ $p < 0.05$ vs. Vhc O_2/O_3, ^{ç} $p < 0.05$ vs. CBD.

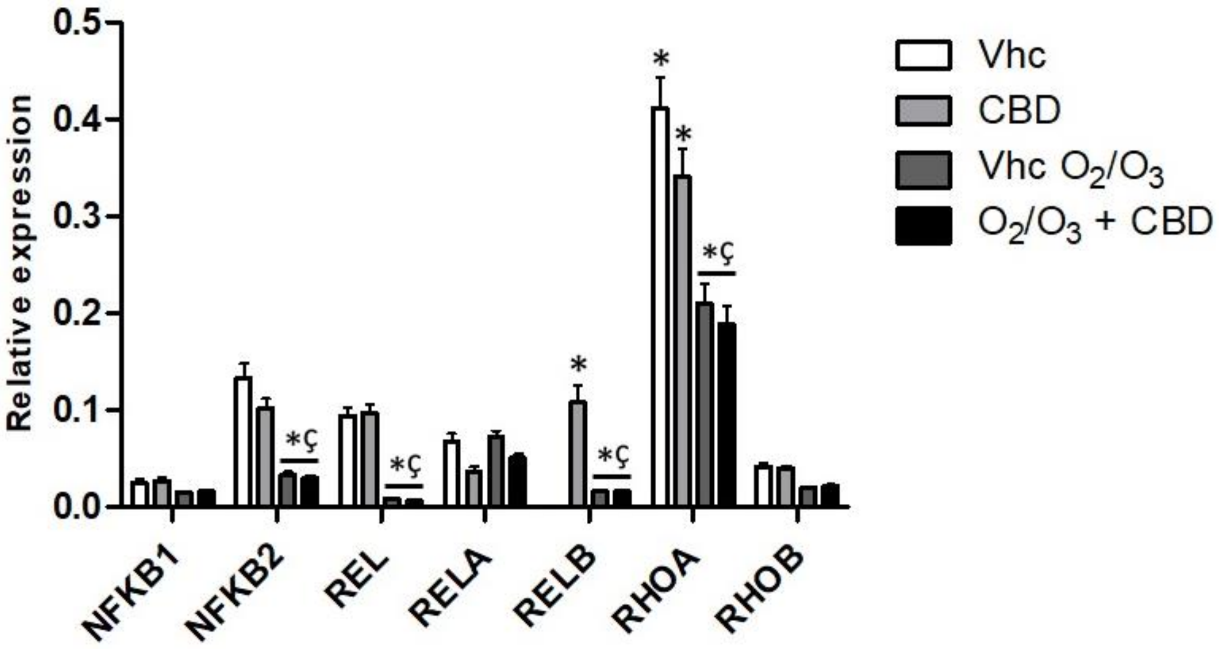

Figure S9. Modulation of NfkB pathways in MiaPaCa-2 cell line. NFKB1, NFKB2, REL, RELA, RELB, RHOA and RHOB mRNA expression was evaluated by qRT-PCR in MiaPaCa-2 cell line, treated with CBD in presence and absence of O_2/O_3. Target mRNA levels were normalized for GAPDH expression. Data are expressed as fold mean ± SE. * $p < 0.05$ vs. Vhc, [#,*] $p < 0.05$ vs. Vhc O_2/O_3, [ç] $p < 0.05$ vs. CBD.

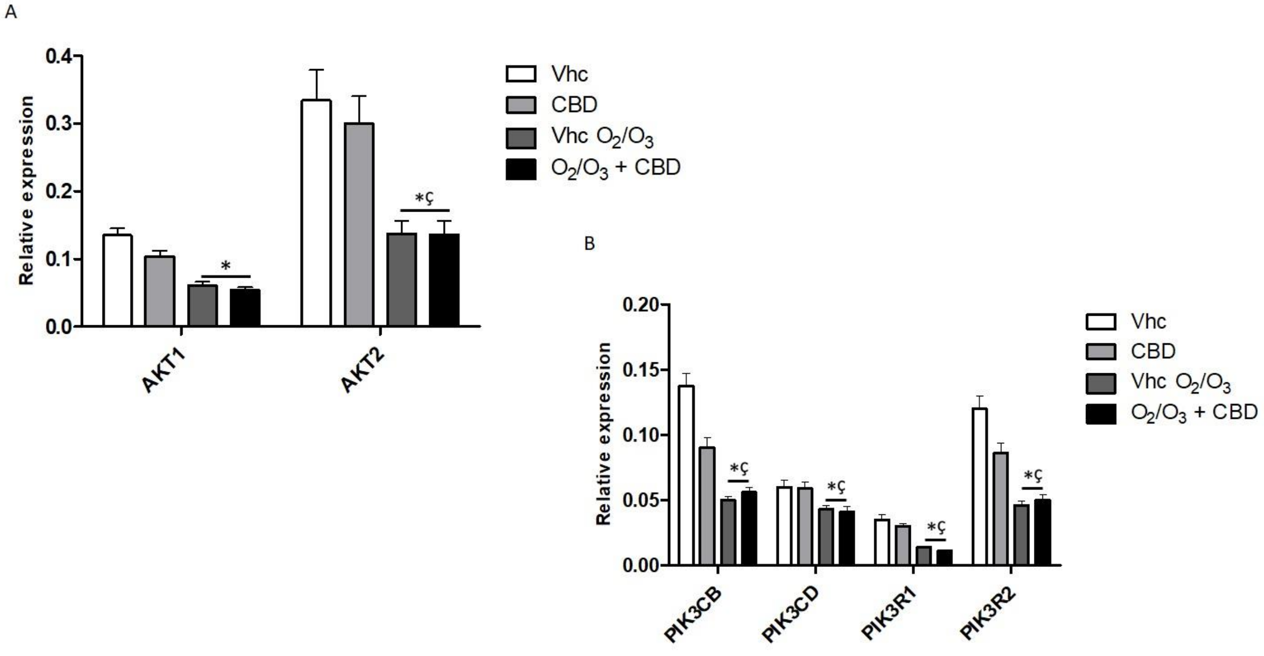

Figure S10. Modulation of PI3K/AKT pathway in MiaPaCa-2 cell line. (**A**) AKT1 and AKT2 mRNA expression and (**B**) PIK3CB, PIK3CD, PIK3R1 and PIK3R2 mRNA expression was evaluated by qRT-PCR in MiaPaCa-2 cell line, treated with CBD in presence and absence of O_2/O_3. Target mRNA levels were normalized for GAPDH expression. Data are expressed as fold mean ± SE. * $p < 0.05$ vs. Vhc, [#,*] $p < 0.05$ vs. Vhc O_2/O_3, [ç] $p < 0.05$ vs. CBD.

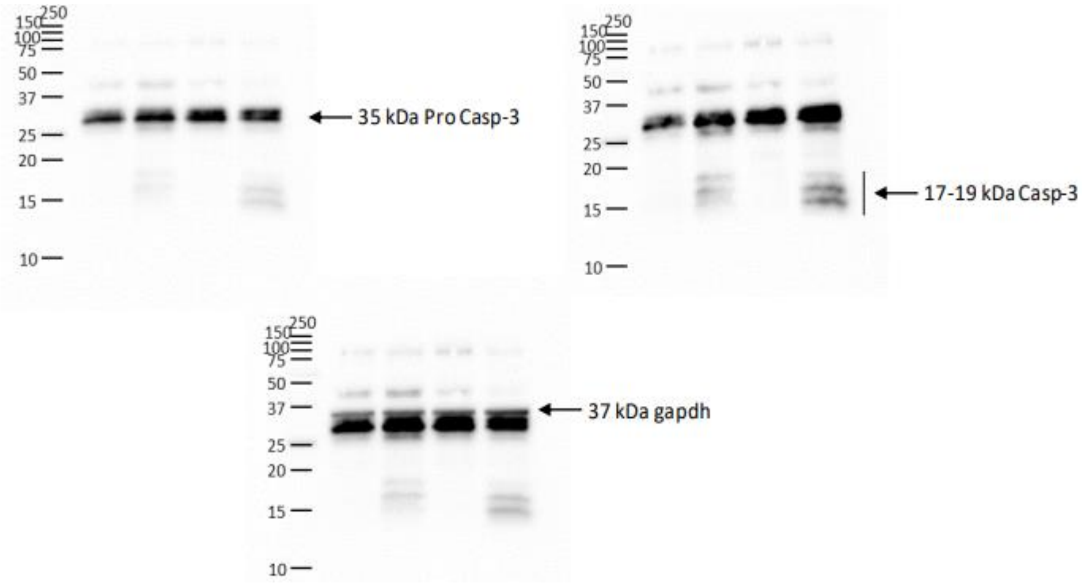
35 kDa Pro Casp-3
17-19 kDa Casp-3
37 kDa gapdh

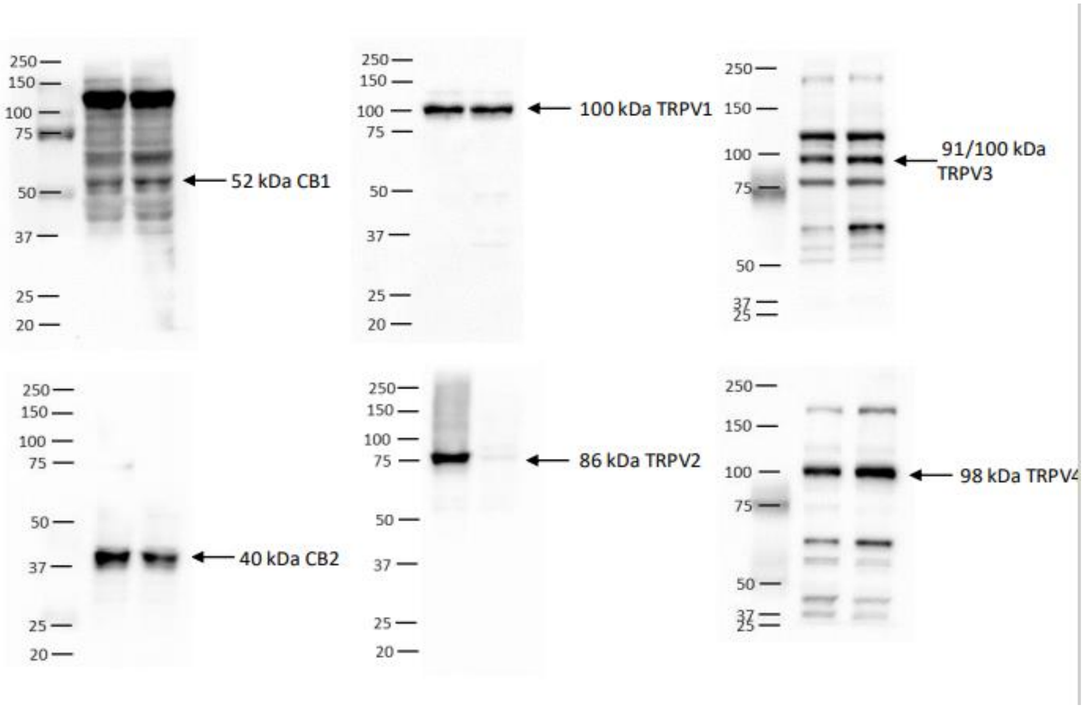
52 kDa CB1
100 kDa TRPV1
91/100 kDa TRPV3
40 kDa CB2
86 kDa TRPV2
98 kDa TRPV4

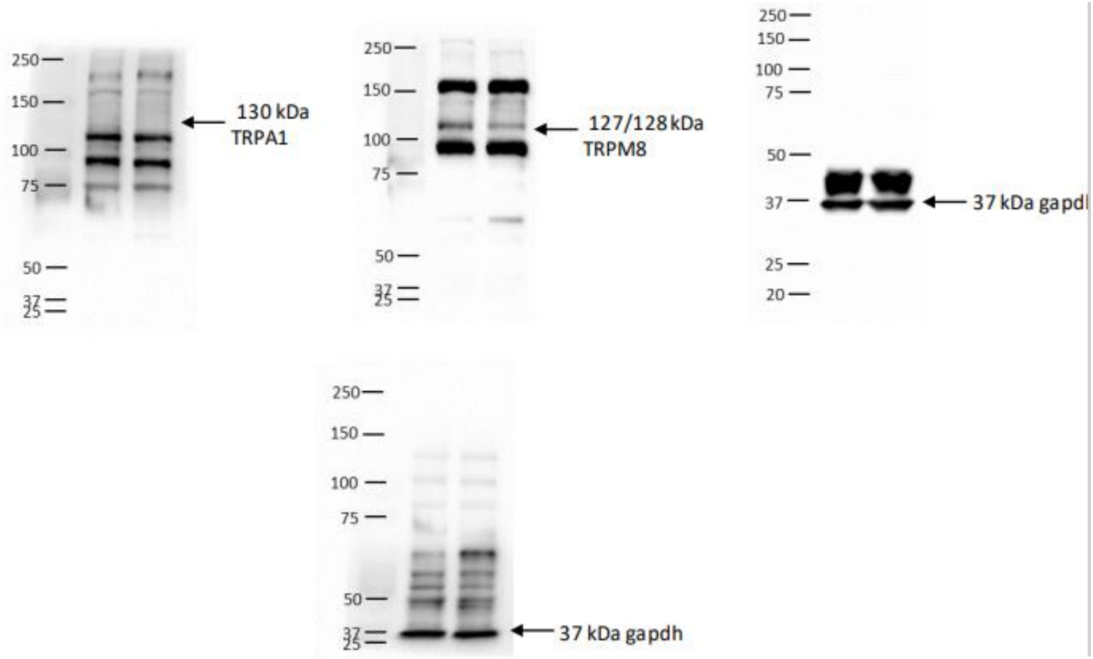

Figure S11. Uncropped Western blot images from Figure 3 and Figure S1.
